# Changes in cultivation parameters impact cytochrome P450 gene transcription in HepaRG cells: implications for *in vitro* toxicological assessments

**DOI:** 10.3389/fphar.2025.1690384

**Published:** 2025-11-03

**Authors:** Kristina Jochum, Veronika Städele, Philip Marx-Stoelting

**Affiliations:** Department of Pesticides Safety, German Federal Institute for Risk Assessment, Berlin, Germany

**Keywords:** cell number, cultivation method, damage, timepoint, PCR, brms, CYP, HepaRG

## Abstract

**Introduction:**

The HepaRG cell line has become a widely used model for liver toxicity testing due to the expression of cytochrome P450 enzymes essential for phase I metabolism of endogenous and exogenous compounds. As variations in expression may pose human health risks, determining CYP interactions of substances is crucial in toxicity assessments. Therefore, the use of human liver cell lines, such as HepaRG, for regulatory hazard assessment requires reproducible and stable CYP enzyme expression, despite possible influencing factors, such as seeding cell number, partial cell monolayer damage, and mRNA extraction timepoint.

**Methods:**

Transcriptional changes of 12 major CYP genes in relation to changes in cultivation parameters were investigated. To this end, HepaRG cells were cultivated according to two different methods and analyzed by RT-qPCR. Cells were seeded at five densities per cultivation method and mRNA was extracted at two timepoints after completion of differentiation, also comparing extracts from undamaged and intentionally damaged cell monolayers.

**Results:**

A Bayesian regression model showed timepoint and cell number to have the most impact on transcription. Transcription was decreased at very high and very low cell numbers over recommended numbers, but this effect was strongly modulated by extraction timepoint, with transcription increasing after two additional weeks in culture. Intentional damage to the cell monolayer had marginal effects on transcription, and no evidence of an effect of cultivation method was found.

**Conclusion:**

In summary, extraction timepoint and seeding cell number are the two critical parameters to consider before initiating a CYP expression experiment with HepaRG cells.

## 1 Introduction

The HepaRG cell line has emerged as a frequently used model in hepatotoxicity studies across various fields, ranging from foundational research and mechanistic studies to regulatory applications (Gerets et al., 2012; Jochum et al., 2024; McGill et al., 2011; Wuerger et al., 2024; Knebel et al., 2022). This hepatocellular carcinoma-derived cell line can differentiate into two distinct cell types, cholangiocyte-like cells and hepatocyte-like cells (Andersson et al., 2012), thus closely resembling mature human hepatocytes (Guillouzo et al., 2007; Guguen-Guillouzo et al., 2010). The use of cryo-preserved HepaRG cells as a model for CYP interaction analyses has been evaluated by the European Union Reference Laboratory for Alternatives to Animal Testing (EURL ECVAM, 2025). While validation and peer review have been completed to a large extent, the process of regulatory implementation is still underway (EURL ECVAM, 2014).

CYP enzymes play a key role in the metabolism of various compounds, and understanding their interactions is essential for assessing potential effects on human health. Physiologically, CYP enzymes are part of the phase I metabolism of substances comprising numerous isoforms with distinct substrate specificities catalyzing specific metabolic reactions. Interactions of xenobiotics with these vital cellular reactions can destabilize a system by potential toxification of substances or reduced detoxification (Esteves et al., 2021; Hodges and Minich, 2015; Zhao et al., 2021). These interactions of substances with CYP enzymes can be analyzed on different levels: enzyme activity, protein expression, mRNA abundance, and receptor activation (Ung et al., 2018; Sinz et al., 2008; Dvorak, 2016; Lee et al., 2024). Here, cell culture techniques with human liver cell lines, such as HepaRG, can be employed (Yamazaki and Tokiwa, 2021; Hart et al., 2010; Bulutoglu et al., 2020; Kim et al., 2024; Foster et al., 2019). For regulatory acceptance and wide-spread application, these cell lines should be easy to use and robustly express CYP enzymes despite potential influencing factors such as seeding cell number, partial damage of the confluent cell monolayer during medium changes, and timepoint of mRNA extraction after completed differentiation. As an additional potential source of variation, different cultivation methods have been proposed for HepaRG to accomplish a stable cell monolayer expressing the main liver enzymes, such as CYPs, close to the levels observed in primary human liver cells (Andersson et al., 2012).

Considering these potential sources of variation, we asked if transcription levels in HepaRG cells of 12 major CYP enzyme genes, i.e., *CYP1A1*, *1A2*, *27A1*, *2B6*, *2C19*, *2C8*, *2C9*, *2D6*, *2E1*, *3A4*, *3A5*, *8B1*, were affected by changes in cultivation parameters. To test this, we analyzed CYP mRNA levels in HepaRG cells cultivated with two different methods, according to the standard protocol for the normal-density process (undifferentiated cells are seeded in culture plates and maintained for 4 weeks to complete differentiation) and according to the high-density process (differentiated cells are seeded in culture plates and maintained for another 2 weeks to yield functional, differentiated cells). Additionally, we seeded cells at five densities per cultivation method and extracted mRNA at two timepoints after completing differentiation, while also comparing extractions from deliberately damaged cell monolayers with those from undamaged cells.

## 2 Materials and methods

### 2.1 Materials

William’s E medium, recombinant human insulin and fetal calf serum (FCS) good forte (catalog no. P40-47500, batch no. P131102) were acquired from PAN-Biotech GmbH (Aidenbach, Germany). Trypsin-EDTA and Penicillin-Streptomycin solution were obtained from Capricorn GmbH (Ebsdorfergrung, Germany). Dimethyl sulfoxide (DMSO) and hydrocortisone-hemisuccinate (HC/HS) were purchased from Sigma-Aldrich (Taufkirchen, Germany).

### 2.2 Cell culture

Undifferentiated HepaRG cells were purchased from Biopredic International (Sant Grégoire, France) and cultivated in 75 cm^2^ flasks under humid conditions at 37 °C and 5% CO_2_ from passage 15 to 20.

For the normal-density cultivation process, cells were grown for 2 weeks in proliferation medium (William’s E medium with 2 mM L-glutamine, supplemented by 10% FCS good forte, 100 U mL^-1^ penicillin, 100 μg mL^-1^ streptomycin, 0.05% human insulin and 50 µM HC/HS) before they were passaged using trypsin-EDTA solution and seeded in 12-well plates at different densities (see Table 1). After an additional 2 weeks in proliferation medium, the medium was changed to differentiation medium (i.e., proliferation medium supplemented by 1.7% DMSO) and cells were cultivated for another 2 weeks.

**TABLE 1:** Seeding cell number and corresponding cell densities per cultivation method.

	Normal-density cultivation method		High-density cultivation method	
	Cell number (10^3^)	Cell density (10^3^ cells cm^-2^)	Cell number (10^3^)	Cell density (10^3^ cells cm^-2^)
Very low	5.2	1.4	130.0	35.6
Low	26.0	7.1	325.0	89.0
Recommended	130.0	35.6	650.0	178.1
High	650.0	178.1	1 300.0	356.2
Very high	3 250.0	890.4	3 250.0	890.4

For the high-density cultivation process, cells were passaged into 75 cm^2^ flasks at a density of 20,000 cells per cm^2^, where they were kept for 2 weeks in proliferation medium followed by 2 weeks in differentiation medium. Then, cells were harvested using trypsin-EDTA and the cell suspension was centrifuged for 3 min at 500 × *g*. Afterwards, the supernatant was aspirated and the cell pellet resuspended in differentiation medium. The resuspended cells were passed through autoclaved 250 µm nylon tissue strainers (Fisher Scientific GmbH, Schwerte, Germany), to break down cell clots. Cells were seeded in 12-well plates at different densities (see Table 1) and kept in differentiation medium for another 2 weeks.

**FIGURE 1:**
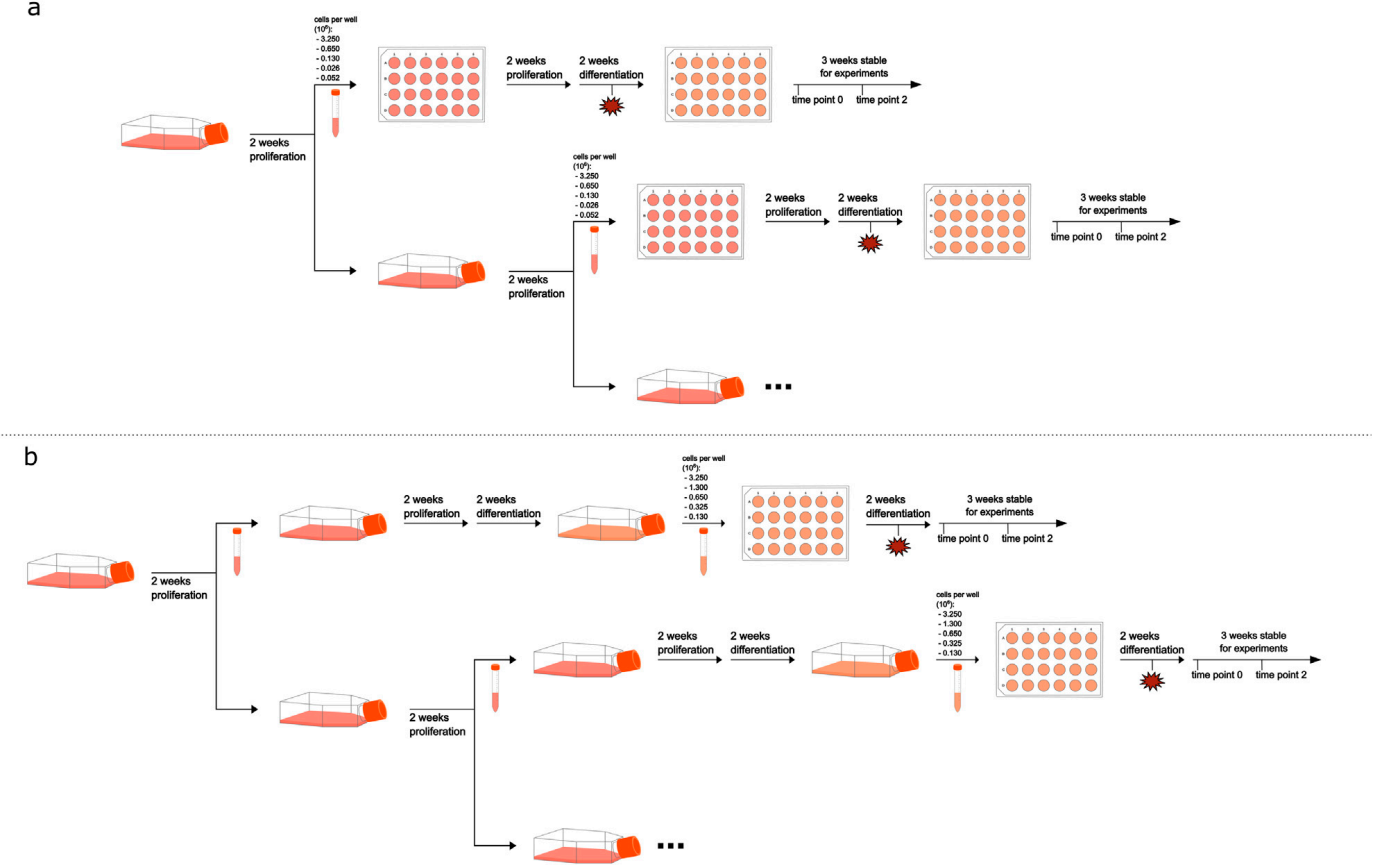
Schematic overview of the cultivation process. Frame **(a)** represents the normal-density cultivation process and frame **(b)** the high-density process. Timepoint of cell monolayer damage is indicated by a red star, applied only to half of the samples.

After the differentiation process was completed, for both cultivation methods, medium was changed to treatment medium (i.e., proliferation medium supplemented by 0.5% DMSO and 2% FCS) and RNA extraction was performed 2 days later, or cells were kept in differentiation medium for another 2 weeks before medium change to treatment medium and subsequent RNA extraction. For investigation of damaged cell monolayers and induced proliferation, parts of the cell monolayer were intentionally aspirated 1 week before the differentiation process was completed (Figure 1). Cell monolayer damage was achieved by intentional aspiration of three, approximately 1 cm long, tracks with the Pasteur pipette used for medium change. Example microscopic pictures taken with a Leica ICC50 HD microscope camera (Wetzlar, Germany) of damaged monolayers can be found in Supplementary Figure S1 (Supplementary File 1).

In total, 40 unique conditions were analyzed in at least three, and a maximum of five, independent experiments. Independent experiments are defined as extractions from different passages where experimental procedures were independently conducted.

### 2.3 Quantitative real-time PCR

Changes in transcription were analyzed with quantitative real-time PCR (RT-qPCR) as previously described (Karaca et al., 2023). In brief, cells were seeded in 12-well plates and RNA extraction was performed with the RNA easy Mini Kit (Qiagen, Venlo, Netherlands) according to the manufacturer’s manual. RNA concentration and purity were determined with a Nanodrop spectrometer (NanoDrop 2000; Thermo Fischer Scientific, Darmstadt, Germany) and 800 ng RNA was used for cDNA synthesis with the High-Capacity cDNA Reverse Transcription Kit (Applied Biosystems, Waltham, MA, USA) with a GeneExplorer 96 (Biozym Scientific GmbH, Hess. Oldendorf, Germany) according to the manufacturer’s protocol. Yield cDNA samples were stored at −20 °C until RT-qPCR was performed with Maxima SYBR Green/ROX Master Mix (Thermo Fisher Scientific, Darmstadt, Germany) according to manufacturer’s protocol. In brief, 20 ng cDNA in 1 µL nuclease-free water was pipetted into each well of a 384-well plate before adding 9 µL master mix (5 µL Maxima SYBR Green/ROX qPCR Master Mix, 0.6 µL each of forward and reverse primers (2.5 µM), and 2.8 µL nuclease-free water). RT-qPCR was performed with an ABI 7900HT Fast Real-Time PCR system instrument (Applied Biosystems, Darmstadt, Germany): activation at 95 °C for 15 min, 40 cycles of 15 s at 95 °C and 60 s at 60 °C, hold at 60 °C for 15 min and default melting curve analysis. Samples were tested for 12 CYP genes, i.e., *CYP1A1*, *1A2*, *27A1*, *2B6*, *2C19*, *2C8*, *2C9*, *2D6*, *2E1*, *3A4*, *3A5*, *8B1*. *GAPDH* and *GUSB* were selected as suitable housekeeping genes and sufficient primer efficiency was verified beforehand. Sequences of all forward and reverse primers can be found in Supplementary Table S1 (Supplementary File 1). Using the Sequence Detection Systems (SDS) software (version 2.4.1), threshold for determining the threshold cycle (C_T_) was set to 0.5, melting curve was checked and manual baseline correction was performed for each gene individually before exportation. For each sample, C_T_s of two technical replicates were averaged and ΔC_T_ was calculated according to Schmittgen and Livak (2008) by subtracting the mean C_T_ of two control genes from the C_T_ of the gene of interest, thus correcting for PCR variability.

### 2.4 Statistical analysis

R 4.4.1 (R Core Team, 2024) and RStudio 2023.9.1 (Posit Team, 2023) were used for data analysis. The R package *brms* was used for Bayesian multilevel modelling (Bürkner, 2017). The data used in this analysis, including sample information regarding cell number, damage to the monolayer, timepoint of mRNA extraction, and cultivation method, as well as calculated ΔC_T_, can be found in the Supplementary File 2. Preliminary analysis indicated that the data were not normally distributed. Therefore, Student-*t* distribution-based linear mixed models with ΔC_T_ as the response variable were constructed. Damage, timepoint, cultivation method and cell number were included as fixed effects, and gene identity as a random intercept, to allow for different levels of transcription among genes. Random slopes were included for all fixed effects within gene identity to allow for the possibility that the influence of a certain variable on gene transcription was dependent on gene identity. In the absence of traditionally used significance levels, the magnitude of the standard deviation in relation to the magnitude of the estimate and how strongly credible intervals overlap zero in addition to model comparison was used to interpret the importance of model terms. Interaction effects were visualized by plotting means and 95% highest posterior density intervals (HPDIs) of draws of the mean of the posterior predictive distribution generated with *posterior_epred* function in *brms* for each gene, as well as medians and 95% HPDIs for pairwise contrasts (differences between levels of a factor) estimated with *emmeans* function in the *emmeans* package (Lenth, 2024) to visualize overall interaction effects. For model comparison, the *loo_compare* function in *brms* was used, which assigns a score of zero to the best fitting model and a difference score relative to this model to all others. Data visualization was done with *ggplot2* package (Wickham, 2016). More detailed explanation of statistical reasoning and approach is provided in Supplementary Information 1 (Supplementary File 1).

## 3 Results

**FIGURE 2:**
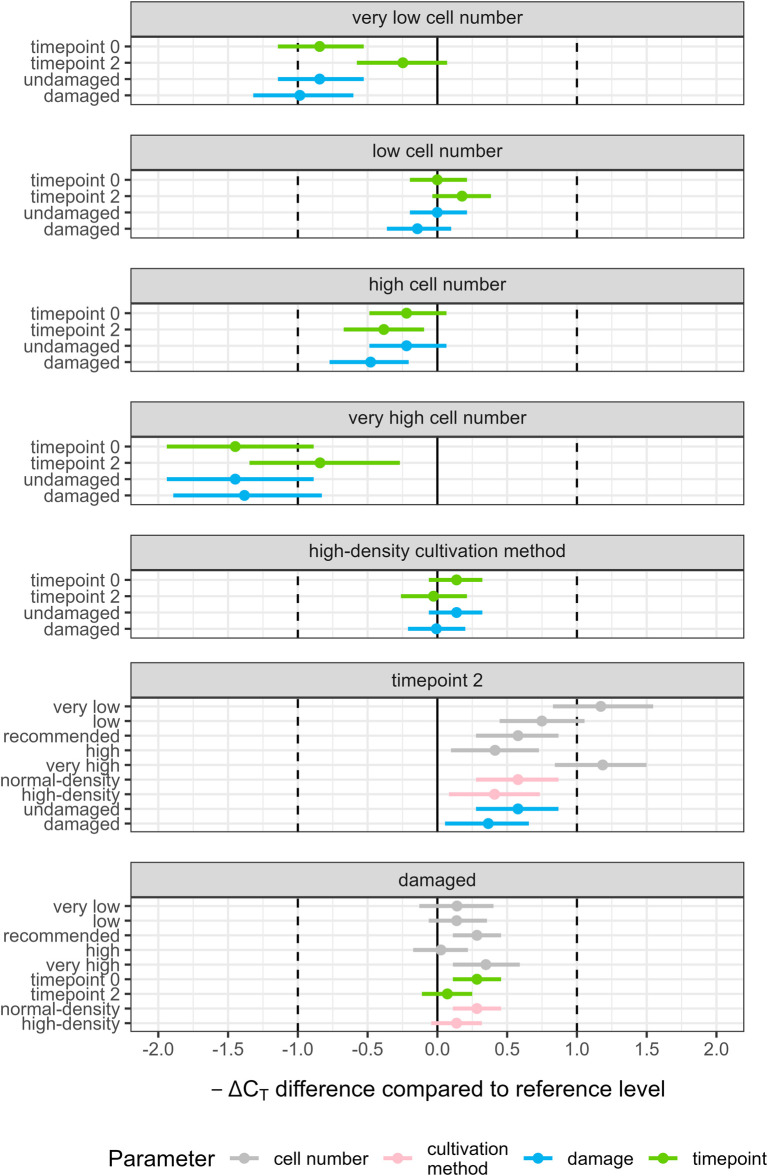
Differences in predicted -ΔC_T_ (contrasts) for all investigated two-way interactions (damage:timepoint, timepoint:cultivation method, damage:cultivation method, damage:cell number, timepoint:cell number) using the data set excluding CYP1A1, 1A2 and 27A1. Gene transcription in HepaRG cells was analyzed in at least three independent experiments using RT-qPCR and relating the results to two housekeeping genes (ΔC_T_). Each panel shows one level of a parameter, with the predicted -ΔC_T_ at the reference level subtracted from the predicted -ΔC_T_: reference levels were recommended cell number, normal-density cultivation method, timepoint 0, and no damage. Estimates within each panel show how the effects are modulated by other parameters. Points are mean posterior predictions; horizontal lines show 95% highest posterior density intervals. All parameters not represented in the investigated two-way interaction were set to their respective reference levels. Vertical solid lines indicate no predicted difference in -ΔC_T_; vertical dashed lines indicate a predicted difference of one -ΔC_T_. A positive difference relates to an increase in transcription under the respective condition and a negative difference to a decrease.

The influence of four parameters on transcription of 12 different CYP enzyme genes in HepaRG cells was investigated. The analysis was conducted using the model with the best model fit to the data which included five two-way interactions: damage:timepoint, timepoint:cultivation method, damage:cultivation method, damage:cell number, timepoint: cell number. Most common results across genes were visualized as pairwise contrasts, i.e., differences in -ΔC_T_ between categories of a parameter (Figure 2). The further a contrast’s point estimate is from zero, the stronger is the estimated effect of the parameter. Non-overlapping HPDIs with zero, or with HPDIs for other contrasts, indicates confidence in the overall effect or its modulation by other parameters.

Changes in cell number, except low numbers, reduced CYP transcription in comparison to the recommended cell number (all point estimates and HPDIs mostly below zero, Figure 2, Panels 1–4). The strongest reduction was seen for very high cell numbers resulting in a reduction of up to −1.45 (95% HPDI: −1.94 to −0.89) -ΔC_T_ over transcription for the recommended number (Figure 2, Panel 4). For very low and very high cell numbers, the effect of cell number was strongly modulated by timepoint in that CYP transcription was reduced less after an additional 2 weeks of cultivation resulting in respective increases for timepoint 2 over timepoint 0 of 1.17 (95% HPDI: 0.83 to 1.55; very low) -ΔC_T_ and 1.18 (95% HPDI: 0.84 to 1.50; very high) -ΔC_T_ (Figure 2, Panel 6). The effect of timepoint at the recommended cell number was 0.58 (95% HPDI: 0.28 to 0.87) -ΔC_T_.

**FIGURE 3:**
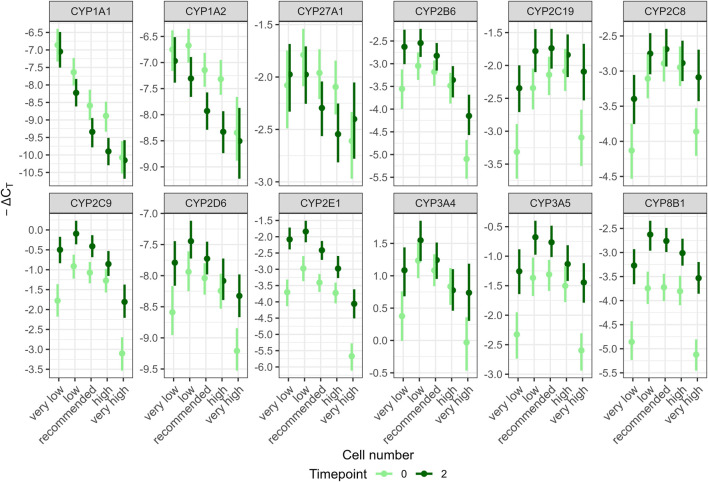
Posterior predictions for the interaction of cell number and timepoint on the gene transcript levels per gene predicted from the fitted model with two-way interactions: damage:timepoint, timepoint:cultivation method, damage:cultivation method, damage:cell number, timepoint:cell number. Gene transcription of 12 CYP genes in HepaRG cells was analyzed in at least three independent experiments using RT-qPCR and relating the results to two housekeeping genes (ΔC_T_). CYP transcript levels were transformed to -ΔC_T_ values by multiplication with −1 to show downregulated gene transcription as lower values. Points represent means, lines represent 95% highest posterior density intervals.

Posterior predictions per gene for the interaction of cell number and timepoint showed an inverted U-shaped relationship for most genes, i.e., transcription was lowest for very low and very high cell numbers and higher for intermediate cell numbers; transcription at timepoint 0 was generally lower than transcription at timepoint 2; at recommended cell numbers, the difference between the timepoints was reduced over more extreme cell numbers (Figure 3). However, one major finding is that these common responses differed considerably for some genes: for *CYP1A1* and *1A2*, transcription tended to decrease with increasing cell numbers and, transcription tended to be higher at the earlier timepoint for *CYP1A1*, *1A2* and *27A1*,. Hence, in order to quantitively describe the most common effects, a modified data set excluding ΔC_T_ values of *CYP1A1*, *1A2* and *27A1* was used to model overall effects (Figure 2). See Supplementary Information 1 for more information on statistical approach (Supplementary File 1). Differences among the remaining genes were quantitative rather than qualitative. For example, at the recommended cell number, differences between timepoints were considerable only for *CYP2E1* and *8B1* (0.98 and 0.97–ΔC_T_, respectively) and much less pronounced for other genes. Per-gene predictions for other interactions are shown in Supplementary Figures S2A–D (Supplementary File 1).

At the global level, no clear effect on gene transcription was observed for cultivation method and low cell numbers (Figure 2, Panels 2 and 5). The effect of damage to the monolayer was inconsistent: damage increased CYP transcription over no damage under some conditions, for example, at the later but not at the earlier timepoint. However, effects were always small (∼0.3−ΔC_T_, Figure 2, Panel 7) and HDPIs were over-lapping with those of other conditions of the same parameter.

## 4 Discussion

The HepaRG cell line has become a frequently used model in hepatotoxicity studies whose robustness and stable expression of CYP enzymes, despite potential influencing factors such as seeding cell number, partial damage of the confluent cell monolayer, and timepoint of analysis, is of great importance. In this study, effects of these parameters on the transcription of 12 CYP genes were observed whereby interactions between the parameters were taken into consideration as well.

Transcription increased at the second timepoint (2 weeks after full differentiation) for all genes except *CYP1A1*, *1A2* and *27A1*, for which this relationship tended to be reversed. The strongest effects were observed for *CYP8B1* and *2E1*. The instructions for both cultivation methods state that cells remain stable for experimental use for at least 3 weeks. Therefore, delaying the extraction by 2 weeks was not expected to have any impact. Savary et al. (2015) assessed the long-term functional stability of HepaRG cells over a 14-day period using a transcriptomics approach and the normal-density cultivation method. They reported that less than 1% of around 13,000 expressed genes were markedly deregulated. Notably, transcription of genes related to xenobiotic metabolism, such as *CYP2A6*, *2C18*, *2C19*, and *3A4*, were upregulated after 3 or 14 days. Similarly, Donato et al. (2022) evaluated the phenotype and functionality of HepaRG cells to demonstrate their suitability for long-term hepatotoxicity assessments and mechanistic studies. HepaRG cells were cultivated according to the normal-density protocol and expression profiles were studied up to 21 days of culture. Among the three CYP genes studied, *CYP1A2* and *2D6* maintained stable expression, whereas *CYP3A4* showed a slight decrease. Additional studies have investigated the stability of HepaRG cells during cultivation and generally concluded an overall stability despite some variability in CYP transcript levels (Anthérieu et al., 2010; Josse et al., 2008). The present study found that the interaction between cell number and timepoint had the strongest impact on model fit. Two weeks after completed differentiation, CYP transcription was particularly increased for very low and very high cell numbers over timepoint 0. However, concentrating on the recommended cell number, this effect is marginal for most genes. Only *CYP2E1* and *8B1* showed differences of more than 1−ΔC_T_ at the recommended cell number (Figure 3). When applying the 2^−ΔΔC_T_^ method proposed by Livak and Schmittgen (2001) for analyzing substance effects on gene transcription, a 2^−ΔΔC_T_^ value greater than 2, or less than 0.5 is usually considered to indicate a meaningful effect, corresponding to a difference of 1−ΔC_T_ between treatment and control. Therefore, if a substance truly has no effect but is applied to cells that are 2 weeks older than those used for the control, the results suggest that a significant effect on gene transcription could still be observed, at least for *CYP2E1* and *CYP8B1*. Consequently, extracting mRNA for multiple replicates at different timepoints, while treating control and treatment groups together, may result in increased experimental variation.

The influence of seeding cell number on transcription is characterized as an inverted U-shape, i.e., very low and very high cell numbers resulted in reduced transcript levels for most genes (except *CYP1A1* and *1A2*). Studies investigating the effects of seeding HepaRG at cell densities lower or higher than recommended are scarce. At high seeding cell numbers, arrest of cell proliferation due to space constraints has been reported (Delarue et al., 2014). Brun et al. (2023) reported early cell enlargement and increased abundance of markers for differentiated cells, such as CYP3A4, in HepaRG cells seeded at high cell numbers, as early as 3 days post-seeding. For lower seeding cell numbers, lower CYP3A4/5 and CYP2B6 activities accompanied by decreases in CYP expression were reported in primary human hepatocytes (Alexandre et al., 2012; LeCluyse et al., 2005; Hamilton et al., 2001). Thus, the observed reduction in CYP expression for high and low seeding cell numbers in the present study may be attributable to stress resulting from space constraints or insufficient cell-cell interactions, respectively. For some genes, i.e., *CYP1A1* and *1A2*, lower cell numbers resulted in increased transcript levels, for *CYP27A1* no clear trend was observed. Studies investigating the cell-density-dependent expression of CYPs in hepatocytes have shown gene-specific variations in mRNA levels (Greuet et al., 1997; Nemoto and Sakurai, 1993). These differences may arise from the distinct regulatory mechanisms of individual CYP genes, as they are influenced by various transcription factors (Honkakoski and Negishi, 2000; Zhao et al., 2021). The differential impact of stress on these transcription factors can contribute to the diverse expression patterns observed among CYP genes (Daskalopoulos et al., 2012). Taken together, when very high and very low cell numbers were analyzed, transcript levels of most analyzed genes differed from the transcript level observed at the recommended cell number by more than 1−ΔC_T_ indicating a substantial effect. However, reduced or increased cell numbers can be detected by microscopic observation early on during the cultivation process. The microscopic pictures in Supplementary Figure S1. Figure 1 illustrate the differences in appearance for undamaged cells at different cell numbers and for both cultivation methods (Supplementary File 1). HepaRG cells differentiate into two morphologically distinct cell types with hepatocyte-like cells appearing as darker clusters throughout the cell monolayer (Guillouzo et al., 2007). At very low and low cell numbers, fewer clusters were observed, while at higher cell number, cells became overcrowded, stacking on top of one another and appearing as dark spots in the microscopic image (Supplementary Figure S1, Supplementary File 1). Therefore, although seeding cell number had substantial impact on gene transcription, regular microscopic monitoring during the cultivation process can help minimize the likelihood of unfavorable cell numbers.

The influence of intentional damage to the confluent cell monolayer was investigated in this study as well. The rationale for examining its impact lies in the possibility that this damage could alter gene transcription, as suggested by hepatectomy studies (Liddle et al., 1989; Marie et al., 1988; Meier et al., 2017; Fujino et al., 2019). These studies have demonstrated changes in CYP mRNA and protein levels, as well as monooxygenase activities, following partial removal of liver tissue. In HepaRG cells, these alterations are thought to result from disrupted cell-cell contact or the ability of HepaRG cells to transdifferentiate, i.e., differentiated cells reverting to a proliferative progenitor state when seeded at low densities (Cerec et al., 2007). In the present study, partial damage to the cell monolayer appeared to affect CYP expression only marginally (Figure 2). Supplementary Figure S1 illustrates the progression of damaged monolayer regions over the two-week investigation period (Supplementary File 1). At timepoint 0, the damaged area began to refill with proliferating cells, which lacked the characteristic clusters of hepatocyte-like cells. By timepoint 2, the damaged regions were fully refilled and appeared differentiated under some conditions. Aspirating parts of the monolayer during medium changes is a common error in cell cultivation and is often a reason for discarding cells before experiments. However, in this study, the observed differences in ΔC_T_ values between damaged and undamaged cells ranged from 0.03 to 0.34 (Supplementary Table S2, Supplementary File 1), suggesting that the effect of such damage on gene transcription may be negligible under most conditions.

Many adaptions to the standard HepaRG cultivation protocol have been proposed, each with its own advantages and disadvantages. Aninat et al. (2006) were among the first to compare two cultivation methods, i.e., low-density and high-density seeding, similar to the approaches used in this study. They observed slightly increased mRNA levels in high-density HepaRG cells, hypothesizing that this was due to a greater proportion of differentiated hepatocyte-like cells. Similarly, Hammour et al. (2022) compared protocols and found that the normal-density cultivation method resulted in higher CYP activities compared to a protocol in which differentiated HepaRG were seeded. However, in both studies, high-density or differentiated-seeded cells were analyzed only 72 h after seeding. Jackson et al. (2016) evaluated cryopreserved, pre-differentiated HepaRG cells and observed that CYP activity decreased within 4 days post-seeding. They reported that these cells only regained their original metabolic competence after approximately 10 days. These findings suggest that differences observed at earlier timepoints may diminish over time, with cells from both methods potentially achieving comparable metabolic performance after extended culture periods. The findings of this study align with that conclusion as no evidence for an effect of cultivation method on CYP transcription under any condition was found.

The present study examined how four cultivation parameters affect the basal transcription of 12 major CYP genes in HepaRG cells; these factors may also influence the outcome of hepatotoxicity studies using this model. The findings provide important insights into how culture conditions may affect the baseline metabolic phenotype of this cell line, but they do not by themselves establish its suitability for studies of hepatotoxicity. Independent studies have shown that differentiated HepaRG cells exhibit transcriptional profiles that are more closely aligned with those of primary human hepatocytes than other hepatic cell lines (Hart et al., 2010; Aninat et al., 2006), supporting their relevance as a model system. Nevertheless, many CYPs are transcriptionally induced by xenobiotics, which is critical for risk assessment purposes. Since the present work was limited to basal expression, future research should address how cultivation conditions modulate CYP transcription in response to known inducers.

## 5 Conclusion

In summary, extraction timepoint and seeding cell number are the two most critical parameters to consider before initiating an experiment with HepaRG cells. While experienced researchers can detect aberrant cell numbers through microscopic observations, the extraction timepoint even for cells seeded at the recommended density strongly affects mRNA levels for some genes. Particular attention should be given to the time of extraction also when testing specific CYP enzymes given that transcription seems to be upregulated early for some genes and late for others. No cultivation method was found to be significantly more robust or susceptible to influencing factors. Damage to the cell monolayer had a marginal impact on the transcription of key CYP genes, suggesting that even less experienced individuals can handle the cells without major issues. However, careful monitoring of cell number throughout the experimental period is essential and should be part of the quality control process.

## Data Availability

The data and R code for data analysis are deposited in the zenodo repository: https://doi.org/10.5281/zenodo.17380238.
