# Supplementary material for: Changes in cultivation parameters impact cytochrome P450 gene transcription in HepaRG cells: implications for in vitro toxicological assessments
Source: Front Pharmacol. 2025 Nov 3;16:1690384. doi: 10.3389/fphar.2025.1690384 (PMC12620486; doi:10.3389/fphar.2025.1690384)
Supplement: Supplementary file 1 [file Supplementaryfile1.docx]

Supplementary Material

**Table of contents**

|  |  | page |
| --- | --- | --- |
| Supplementary Information 1 | Statistical reasoning and approach | 3 |
| Supplementary Figure 1 | Microscopic observation of HepaRG cells at each analyzed condition | 6 |
| Supplementary Table 1 | Primer sequences | 7 |
| Supplementary Figure 2A-D | Effects of the interactions on the gene transcript levels per gene | 8 |
| Supplementary Table 2 | Differences in predicted ∆C_T_, and lower and upper limits of a 95 % HPDI for all investigated two-way interactions | 12 |
| Supplementary Table 3 | elpd difference score and standard error of the comparison of the fitted models with three-way interactions or all resulting two-way interactions | 14 |
| Supplementary Figure 3 | Graphical representations of elpd difference score and standard error for the comparison of the fitted models with three-way interactions or all resulting two-way interactions | 15 |
| Supplementary Table 4 | elpd difference score and standard error of the comparison of the fitted model with or without different two-way interactions | 16 |
| Supplementary Figure 4 | Graphical representation of elpd difference score and standard error of the comparison of the fitted model with different two-way interactions | 17 |
| Supplementary Table 5 | Estimates, standard deviations (SD), and lower and upper limits of 95 % credible intervals (l-95 % CI, u-95 % CI) of the fitted model including the two-way interactions | 18 |
| Supplementary Figure 5 | Posterior predictive check of the type “dens_overlay_grouped” for the fitted model with two-way interactions | 19 |
| Supplementary Table 6 | elpd difference score and standard error of the comparison of the fitted model from the data set excluding CYP1A1, CYP1A2 and CYP27A1 with or without different two-way interactions | 20 |
| Supplementary Figure 6 | Graphical representation of elpd difference score and standard error of the comparison of the fitted model from the data set excluding CYP1A1, CYP1A2 and CYP27A1 with or without different two-way interactions | 21 |
| Supplementary Table 7 | Estimate, estimated error, and lower and upper limits of 95 % credible intervals (l-95 % CI, u-95 % CI) of the fitted model from the data set excluding CYP1A1, CYP1A2 and CYP27A1 | 22 |

**Supplementary Information 1:** Statistical reasoning and approach.

In a first step of the analysis, four obvious outliers were visually identified as ΔC_T_ above 13 and excluded from the data set. ΔC_T_ values in the final data set ranged from 12.3 to -2.5. All four investigated parameters were discrete variables and transformed into factors: damaged/undamaged, timepoint 0/timepoint 2, normal-density cultivation method/high-density cultivation method; cell number was classified as ‘very low’, ‘low’, ‘recommended’, ‘high’, and ‘very high’ instead of using cell number as a continuous variable. This classification was necessary to accurately assess the effects of varying cell numbers. In this analysis, cell numbers corresponding to each category varied between the cultivation methods as seeding cell numbers were selected based on the ‘recommended’ cell number and are expressed as multiples of this baseline. Thus, treating cell number as a categorical variable prevents misinterpretation of data. For instance, it avoids modeling ‘high’ cell numbers in the normal-density method (650 000 cells) as equivalent to ‘recommended’ cell numbers in the high-density method (650 000 cells), acknowledging the procedural differences between methods. In the normal-density cultivation process, proliferating cells grow for an additional 2 weeks before differentiation. Consequently, fewer cells are initially seeded compared to the high-density process (‘recommended’: 130 000 vs 650 000, respectively), but extracted cell numbers are similar, as both methods result in confluent cell monolayers.

The following reference levels were set for the analyzed parameters: undamaged, timepoint 0, normal-density cultivation method, ‘recommended’ cell number. Distinct effects were anticipated for each parameter. Damage and reduced cell numbers were expected to decrease CYP expression, whereas there was no clear prediction for large cell numbers. The cultivation method was expected to influence CYP expression and other parameters, while the extraction timepoint was not expected to have an impact, as both cultivation protocols should ensure stable cells for at least three weeks.

We initially included two three-way interactions (cell number:damage:timepoint and damage:timepoint:cultivation method). We then compared this model, to two models including only one of the three-way interactions each and the remaining two-way interactions, and to a model only including the two-way interactions to identify whether the model with two three-way interactions improved model fit over simpler models. We used *loo_compare* in *brms* for model comparison. The function computes a formal difference score between the models based on the information criterion. The output ranks models by fit, with the best model receiving a score of zero and all others receiving a difference score relative to it. The function *pp_check* was applied to verify the model performance. A three-way interaction between the parameters cell number, damage and timepoint is theorized as the influence of damage on gene transcription levels could depend on the cell number, as reduced cell numbers could result in more proliferating cells at the time of damage and thus being more susceptible to damage. The effects of damage could be compensated for at a later timepoint by full regrowth of damaged monolayer parts, thus resulting in the three-way interaction cell number:damage:timepoint. Another possible three-way interaction is damage, timepoint and cultivation method. Here, the effect of damage could depend on the cultivation method as cells seeded when they are already differentiated could be more robust against damage. However, a later timepoint could overcome the effects of damage especially for cells seeded in their undifferentiated state. The other possible three-way interactions (cell number:damage:cultivation method and cell number:timepoint:cultivation method) seemed unlikely. Although the effect of damage could depend on the cell number, this should be independent of the cultivation method and although the effect of damage could vary between cultivation methods, this should be independent of the cell number. In addition, the instructions for both cultivation methods state that after completed differentiation, cells are stable for up to three weeks. Thus, transcript levels of undamaged cells should not be influenced by the extraction timepoint. Lastly, although a later timepoint could overcome the effects of lower starting cell number this should be independent of the cultivation method.

From the two initially hypothesized three-way interactions, none improved model fit over a model including all resulting two-way interactions: damage:timepoint, timepoint:cultivation method, damage:cultivation method, damage:cell number, timepoint:cell number (Supplementary Table 3 and Supplementary Figure 3). Possible further simplification of the model containing all two-way interactions was investigated by comparing it to models excluding one two-way interaction at a time, to identify the interaction terms that most strongly explained the data, to a model without any interactions and to an intercepts-only model. This comparison showed that the interaction between cell number and timepoint had the strongest effect on model fit (elpd difference score: -36.6 ± 11.6; Supplementary Table 4 and Supplementary Figure 4). Hence, a model only correcting for this interaction was also included in the comparison. The model excluding the interaction of cell number and damage fit the data better than the second-best fitting model including all two-way interaction (elpd difference score: ‑2.7 ± 3.8; Supplementary Table 4 and Supplementary Figure 4). However, looking at the standard error, it did not perform substantially better than the all-two-way model. In any case, the all-two-way model outperformed the model without any interaction and the model with only the interaction between cell number and timepoint (Supplementary Table 4 and Supplementary Figure 4). We therefore decided to present the results for the model including all two-way interactions. Supplementary Table 5 shows model coefficients and associated uncertainties for the model including all two-way interactions.

Posterior predictive checks indicated that the model predicted the data reasonably well for most genes with the worst prediction being for *CYP1A1* (Supplementary Figure 5). Standard deviations of the random effect coefficients indicated that there was considerable variance among genes for at least some terms (Supplementary Table 5); we therefore first plotted predictions by gene (Figure 3, Supplementary Figure 2A-D). Plots of posterior predictions for single genes showed that CYP1A1, CYP1A2 and CYP27A1 mRNA levels differed from the others with regard to cell number and timepoint (Figure 3). Thus, a modified data set excluding ΔC_T_ values of CYP1A1, 1A2 and 27A1 was constructed to model the more commonly expected overall effect. The same procedure as described above for all two-way interactions was followed to determine the model that best fit the data. Results were similar to the model including all genes (Supplementary Table 6, Supplementary Figure 6). Hence, the all-two-way model was further used to model global effects. See Supplementary Table 7 for model coefficients and associated uncertainties for the model including all two-way interactions but excluding CYP1A1, CYP1A2 and CYP27A1 from the data.

In the absence of traditionally used significance levels, the importance of model terms should be interpreted according to the magnitude of an estimate and its associated uncertainty, i.e., the magnitude of the standard deviation in relation to the magnitude of the estimate and how strongly credible intervals overlap zero. For interactions however, effects are more difficult to interpret from coefficients alone, as estimates and uncertainties of interaction terms and single variable terms combine. We therefore visualized interaction effects by plotting means and 95 % highest posterior density intervals (HPDIs) of draws of the mean of the posterior predictive distribution generated with *posterior_epred* for each gene, as well as medians and 95 % HPDIs for pairwise contrasts (differences between levels of a factor) estimated with *emmeans* in the *emmeans* package (Lenth 2024) to visualize overall interaction effects. To facilitate interpretation, posterior predictions are displayed as -ΔC_T_ by multiplication with -1. Contrasts are calculated as predicted ΔC_T_ subtracted from the predicted ΔC_T_ at the reference level, being equal to subtracting the predicted -ΔC_T_ at the reference level from the predicted -ΔC_T_, as displayed in Figure 2. For all predictions, variables not involved in the interactions were set to their reference categories.


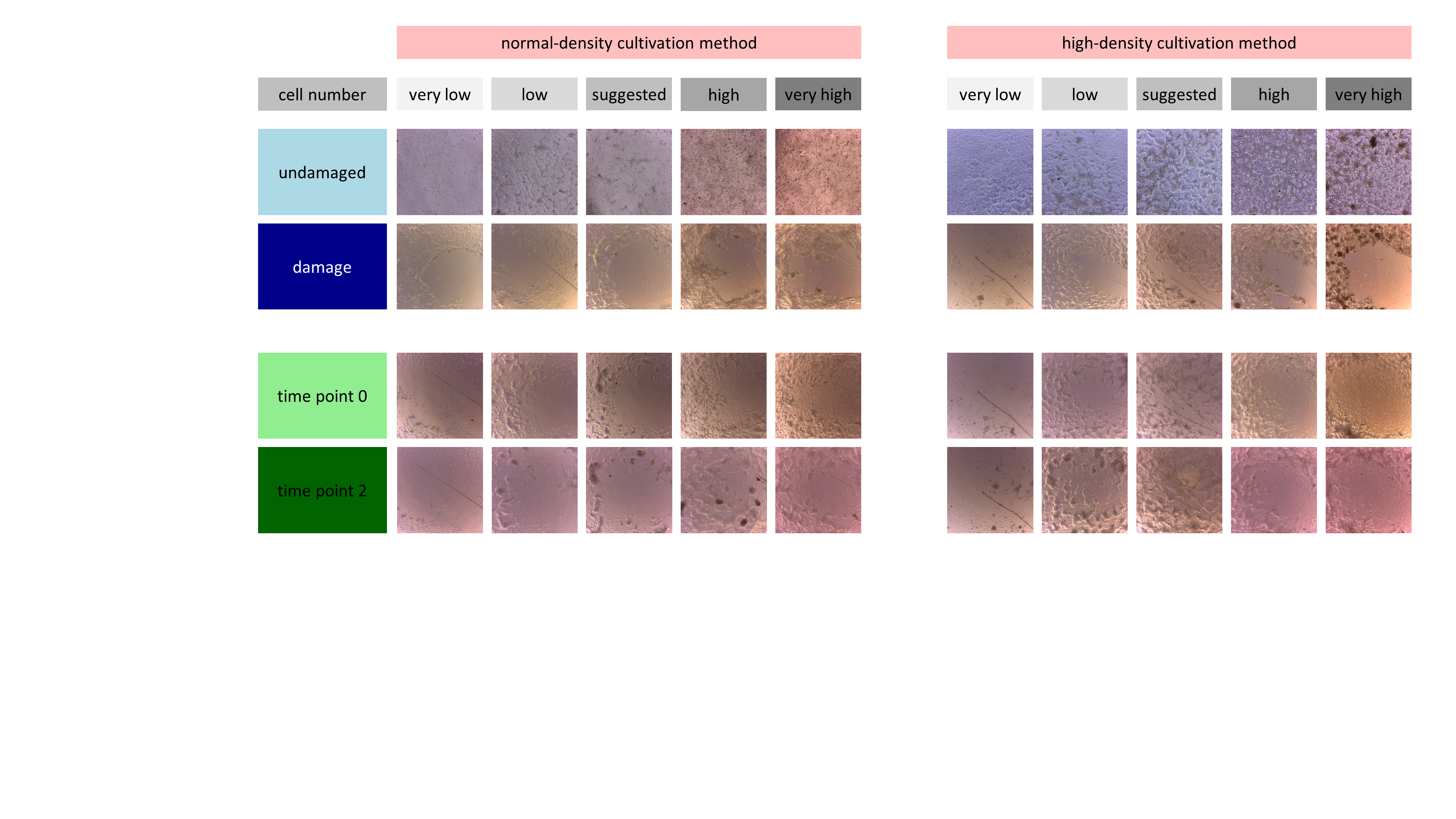


**Supplementary Figure** 1: Microscopic observation (10X) of HepaRG cells at each analyzed condition with a Leica ICC50 HD microscope camera.

**Supplementary Table** **1**: Primer sequences. fw, forward; rv, reverse.

| CYP1A1 fw | 5’-ACC CTG AAG GTG ACA GTT CC-3’ |
| --- | --- |
| CYP1A1 rv | 5’-TCT TGG AGG TGG CTG AGG TA-3’ |
| CYP1A2 fw | 5’-CCT CGC TAC CTG CCT AAC CC-3’ |
| CYP1A2 rv | 5’-CCC GGA CAC TGT TAT TGT CA-3’ |
| CYP2B6 fw | 5’-TTC GGC GAT TCT CTG TGA CC-3’ |
| CYP2B6 rv | 5’-ATG AGG GCC CCC TTG GAT-3’ |
| CYP2C19 fw | 5’-CCT GGA ACG CAT GGT GGT-3’ |
| CYP2C19 rv | 5’-TCC ATT GCT GAA AAC GAT TCC AAA T-3’ |
| CYP2C8 fw | 5’-CCT CCTC ATC AAA TCT TCC CAT T-3’ |
| CYP2C8 rv | 5’-GCA GTG ACC TGA ACA ACT CTC C-3’ |
| CYP2C9 fw | 5’-AAA TGG AGA AGG AAA AGC ACA ACC-3’ |
| CYP2C9 rv | 5’-TCA ACT GCA GTG TTT TCC AAG C-3’ |
| CYP2D6 fw | 5’-GCC TCC CTG CCT TTC TCA GCA G-3’ |
| CYP2D6 rv | 5’-ATG GGC TCA CCA GGA AAG CAA A-3’ |
| CYP2E1 fw | 5’-CAT GAG ATT CAG CGG TTC ACT-3’ |
| CYP2E1 rv | 5’-GGT GTC TCG GGT TGC TTC A-3’ |
| CYP3A4 fw | 5’-TCA CAA ACC GGA GGC CTT TT-3’ |
| CYP3A4 rv | 5’-TGG TGA AGG TTG GAG ACA GC-3’ |
| CYP3A5 FW | 5’-GCC CAA TAA GGC ACC ACC TA-3’ |
| CYP3A5 rv | 5’-CCA CCA TTG ACC CTT TGG GA-3’ |
| CYP8B1 fw | 5’-GGG AGG TTC TTT GCA CTC AG-3’ |
| CYP8B1 rv | 5’-TAG TGG TGT GTC AGG GTC-3’ |
| CYP27A1 fw | 5’-TCC GAG AAA CGC ATT GGC TG-3’ |
| CYP27A1 rv | 5’-GGA GGA AGG TGG CAT AGA GTG-3’ |
| GAPDH fw | 5‘-TTA AAAA GCA GCC CTG GTG AC-3’ |
| GAPDH rv | 5’-CTC TGC TCC TCC TGT TCG AC-3’ |
| GUSB fw | 5’-CAC CAG GGA CCA TCC AAT AC-3’ |
| GUSB rv | 5’-ATG TAG GTG GTG GGT GTC GT-3’ |

A

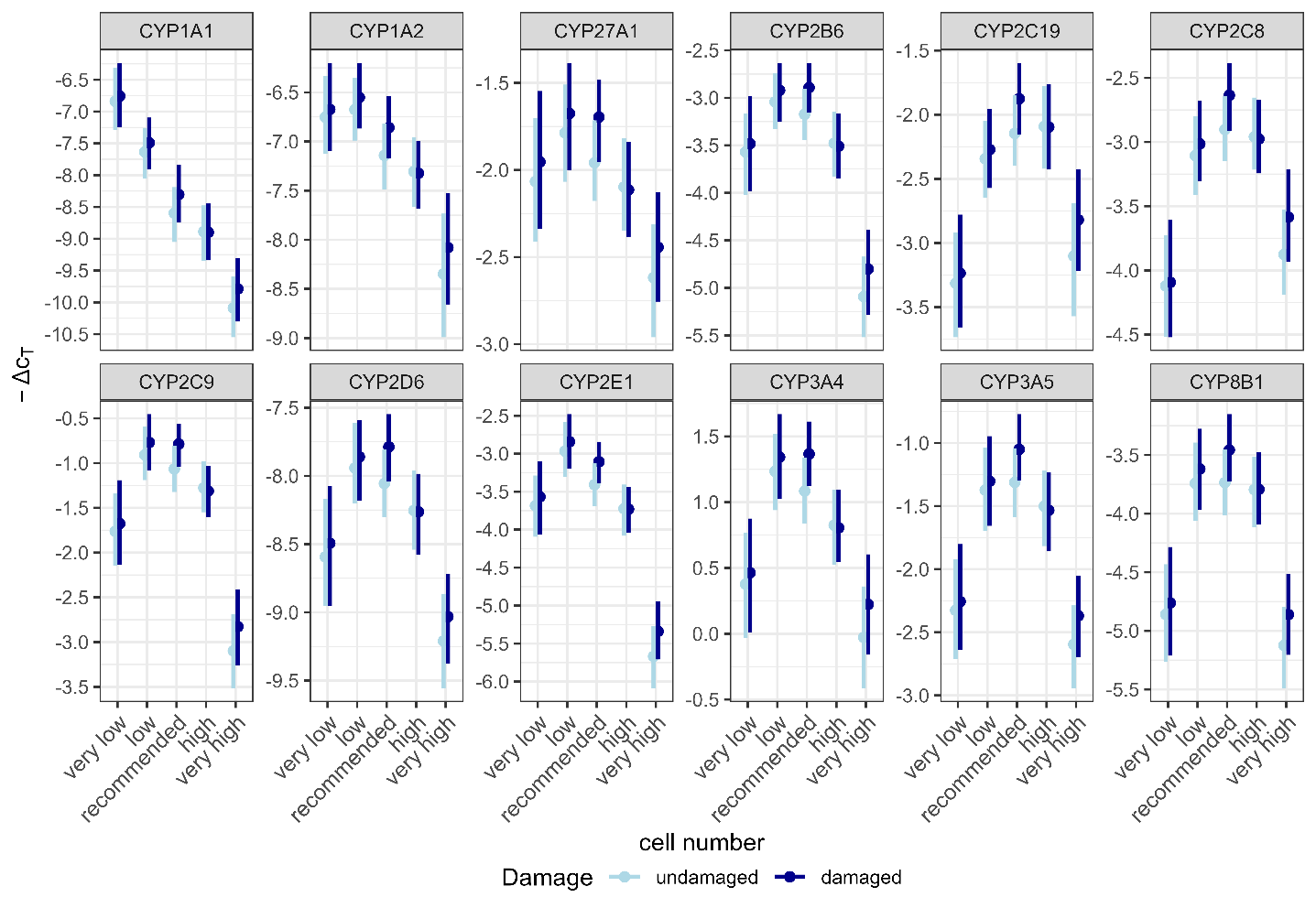


B

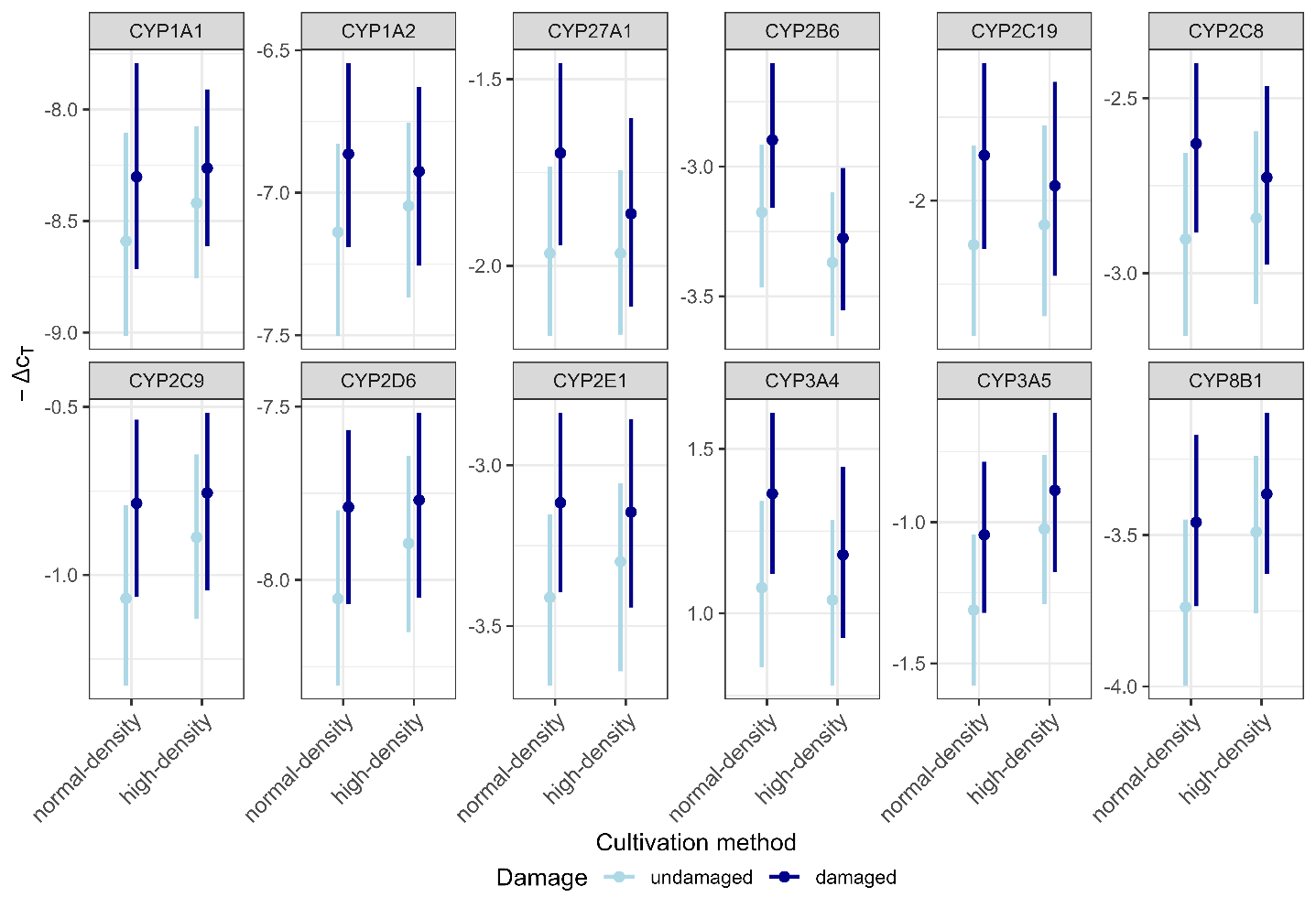


C

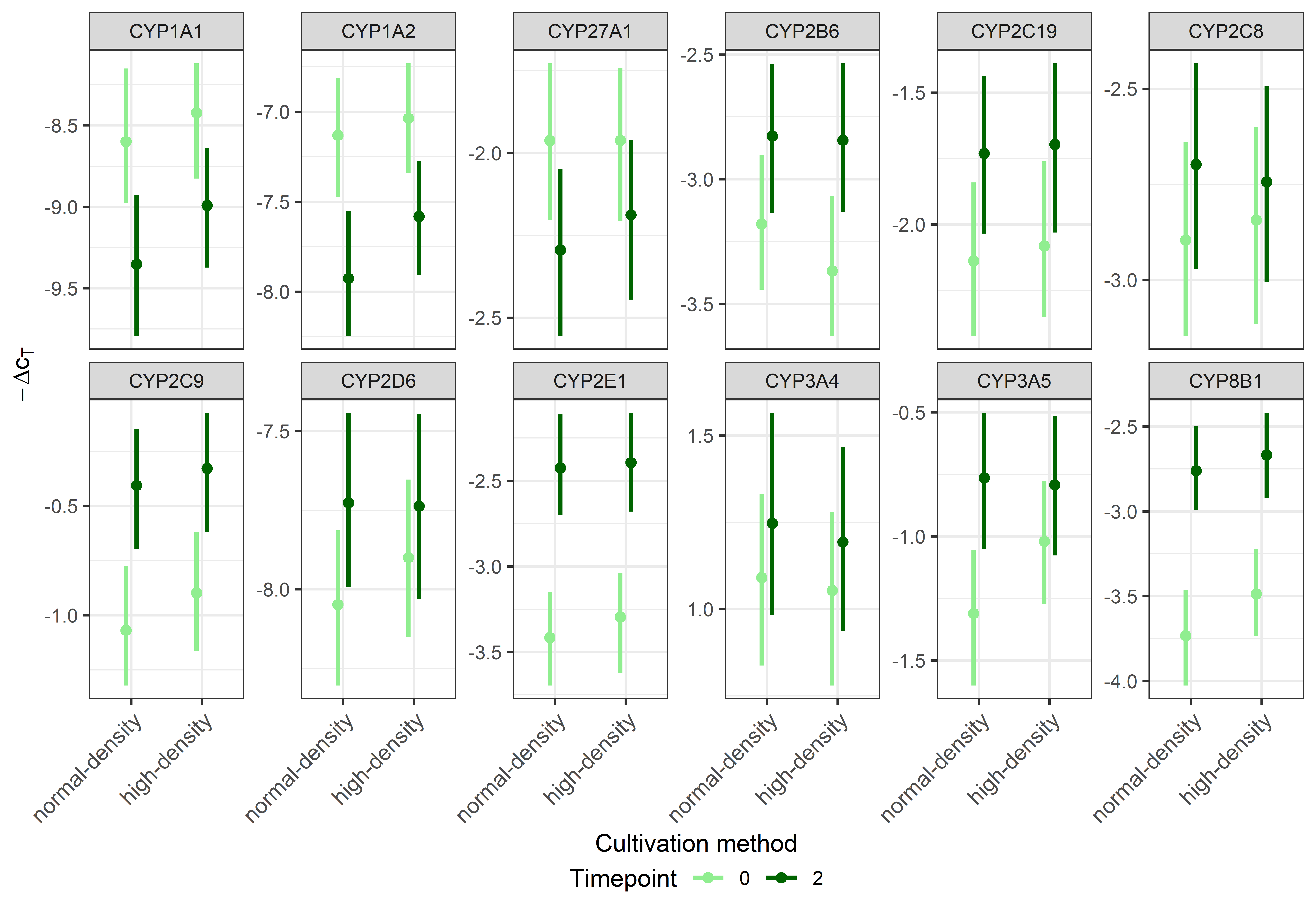


D

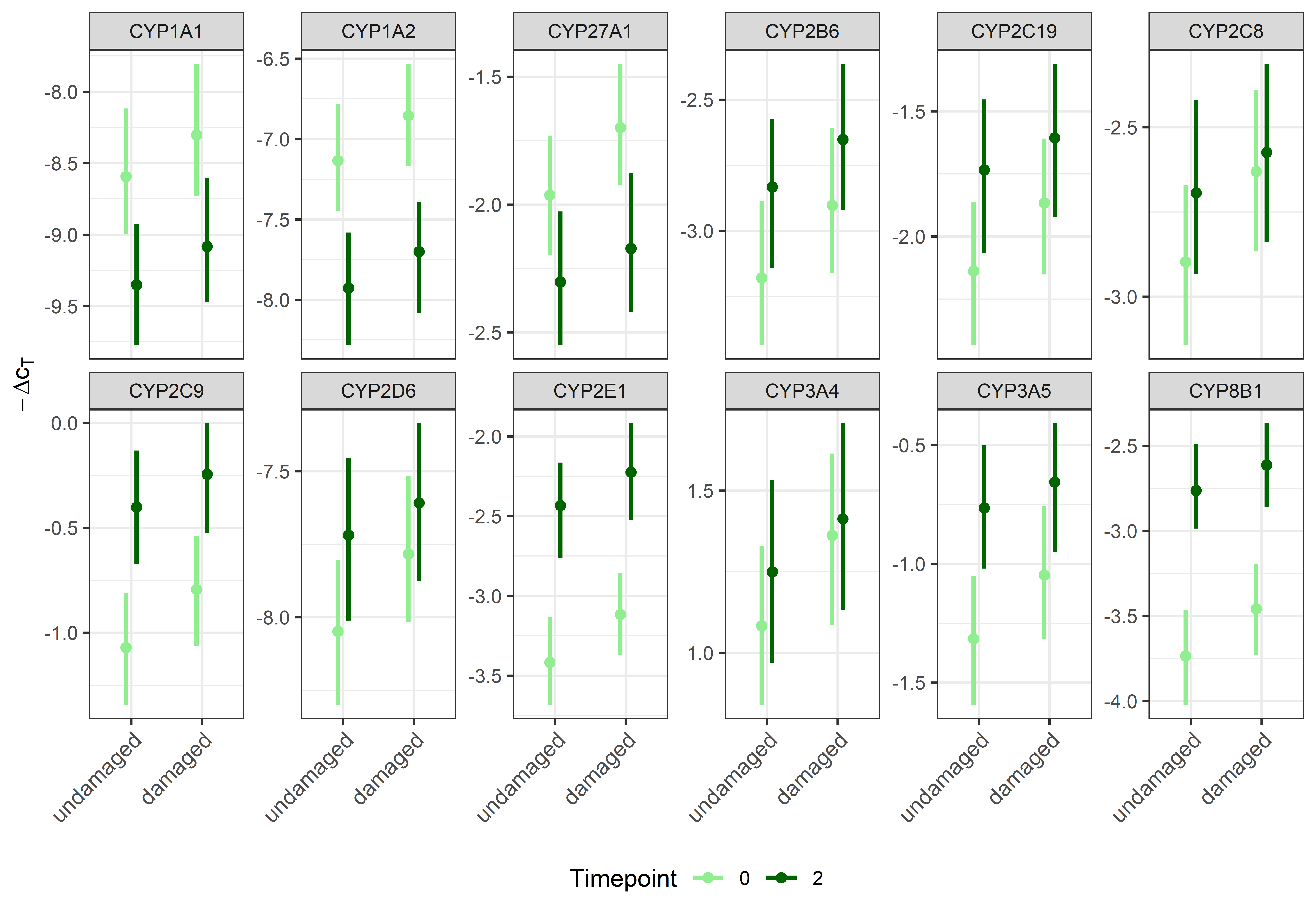


**Supplementary Figure** **2**: Effects of the interactions on the gene transcript levels per gene predicted from the fitted model with two-way interactions: damage:timepoint, timepoint:cultivation method, damage:cultivation method, damage:cell number, timepoint:cell number. Transcription of 12 CYP genes in HepaRG cells was analyzed in at least three independent experiments using RT-qPCR and relating the results to two housekeeping genes (∆C_T_). Transcript levels were transformed to -ΔC_T_ values by multiplication with ‑1 to represent down-regulated gene transcription as lowered values. Points represent means, lines represent 95 % highest posterior density intervals. A: damage:cell number, B: damage:cultivation method, C: timepoint:cultivation method, D: damage:timepoint.

**Supplementary Table** **2**: Differences in predicted ∆C_T_ (contrasts), lower and upper limits of a 95 % HPDI for all investigated two-way interactions (damage:timepoint, timepoint:cultivation method, damage:cultivation method, damage:cell number, timepoint:cell number) using the data set excluding CYP1A1, 1A2 and 27A1. All parameters not represented in the investigated two-way interaction were set to their respective reference levels. Gene transcription in HepaRG cells was analyzed in at least three independent experiments using RT-qPCR and relating the results to two housekeeping genes (∆C_T_).

| Parameter | Level | Contrast | Contrast | lower HPD limit | upper HPD limit |
| --- | --- | --- | --- | --- | --- |
| cell number | recommended | timepoint 0 -  timepoint 2 | 0.58 | 0.28 | 0.87 |
| cell number | very low | timepoint 0 -  timepoint 2 | 1.17 | 0.83 | 1.55 |
| cell number | low | timepoint 0 -  timepoint 2 | 0.75 | 0.45 | 1.05 |
| cell number | high | timepoint 0 -  timepoint 2 | 0.41 | 0.10 | 0.73 |
| cell number | very high | timepoint 0 -  timepoint 2 | 1.18 | 0.84 | 1.50 |
| timepoint | timepoint 0 | recommended -  very low cell number | -0.84 | -1.14 | -0.53 |
| timepoint | timepoint 0 | recommended -  low cell number | 0.00 | -0.20 | 0.21 |
| timepoint | timepoint 0 | recommended -  high cell number | -0.22 | -0.49 | 0.07 |
| timepoint | timepoint 0 | recommended -  very high cell number | -1.45 | -1.94 | -0.89 |
| timepoint | timepoint 2 | recommended -  very low cell number | -0.25 | -0.58 | 0.07 |
| timepoint | timepoint 2 | recommended -  low cell number | 0.18 | -0.04 | 0.38 |
| timepoint | timepoint 2 | recommended -  high cell number | -0.38 | -0.67 | -0.10 |
| timepoint | timepoint 2 | recommended -  very high cell number | -0.84 | -1.35 | -0.27 |
| cell number | recommended | undamaged -  damaged | 0.28 | 0.11 | 0.46 |
| cell number | very low | undamaged -  damaged | 0.14 | -0.13 | 0.40 |
| cell number | low | undamaged -  damaged | 0.14 | -0.06 | 0.36 |
| cell number | high | undamaged -  damaged | 0.03 | -0.17 | 0.22 |
| cell number | very high | undamaged -  damaged | 0.35 | 0.11 | 0.59 |
| damage | undamaged | recommended -  very low cell number | -0.84 | -1.14 | -0.53 |
| damage | undamaged | recommended -  low cell number | 0.00 | -0.20 | 0.21 |
| damage | undamaged | recommended -  high cell number | -0.22 | -0.49 | 0.07 |
| damage | undamaged | recommended -  very high cell number | -1.45 | -1.94 | -0.89 |
| damage | damaged | recommended -  very low cell number | -0.99 | -1.32 | -0.60 |
| damage | damaged | recommended -  low cell number | -0.14 | -0.36 | 0.10 |
| damage | damaged | recommended -  high cell number | -0.48 | -0.77 | -0.21 |
| damage | damaged | recommended -  very high cell number | -1.38 | -1.89 | -0.83 |
| cultivation method | normal-density | timepoint 0 -  timepoint 2 | 0.58 | 0.28 | 0.87 |
| cultivation method | high-density | timepoint 0 -  timepoint 2 | 0.41 | 0.08 | 0.73 |
| timepoint | timepoint 0 | normal-density *-*  high-density cultivation method | 0.14 | -0.06 | 0.32 |
| timepoint | timepoint 2 | normal-density *-*  high-density cultivation method | -0.03 | -0.26 | 0.21 |
| damage | undamaged | timepoint 0 -  timepoint 2 | 0.58 | 0.28 | 0.87 |
| damage | damaged | timepoint 0 -  timepoint 2 | 0.36 | 0.05 | 0.66 |
| timepoint | timepoint 0 | undamaged -  damaged | 0.28 | 0.11 | 0.46 |
| timepoint | timepoint 2 | undamaged -  damaged | 0.07 | -0.11 | 0.25 |
| cultivation method | normal-density | undamaged -  damaged | 0.28 | 0.11 | 0.46 |
| cultivation method | high-density | undamaged -  damaged | 0.14 | -0.04 | 0.32 |
| damage | undamaged | normal-density *-*  high-density cultivation method | 0.14 | -0.06 | 0.32 |
| damage | damaged | normal-density *-*  high-density cultivation method | -0.01 | -0.21 | 0.20 |

**Supplementary Table** 3: elpd difference score and standard error of the comparison of the fitted models with three-way interactions or all resulting two-way interactions using brms package and loo_compare function.

| Model | elpd_diff | se_diff |
| --- | --- | --- |
| all two-way interactions:  damage:timepoint; timepoint:cultivation method;  damage:cultivation method; damage:cell number;  timepoint:cell number | 0.00 | 0.00 |
| three-way interaction:  damage:timepoint:cultivation method | -1.55 | 0.95 |
| three-way interaction:  cell number:damage:timepoint | -4.25 | 2.95 |
| two three-way interactions:  damage:timepoint:cultivation method  cell number:damage:timepoint | -5.92 | 3.04 |

: indicates interaction of variables


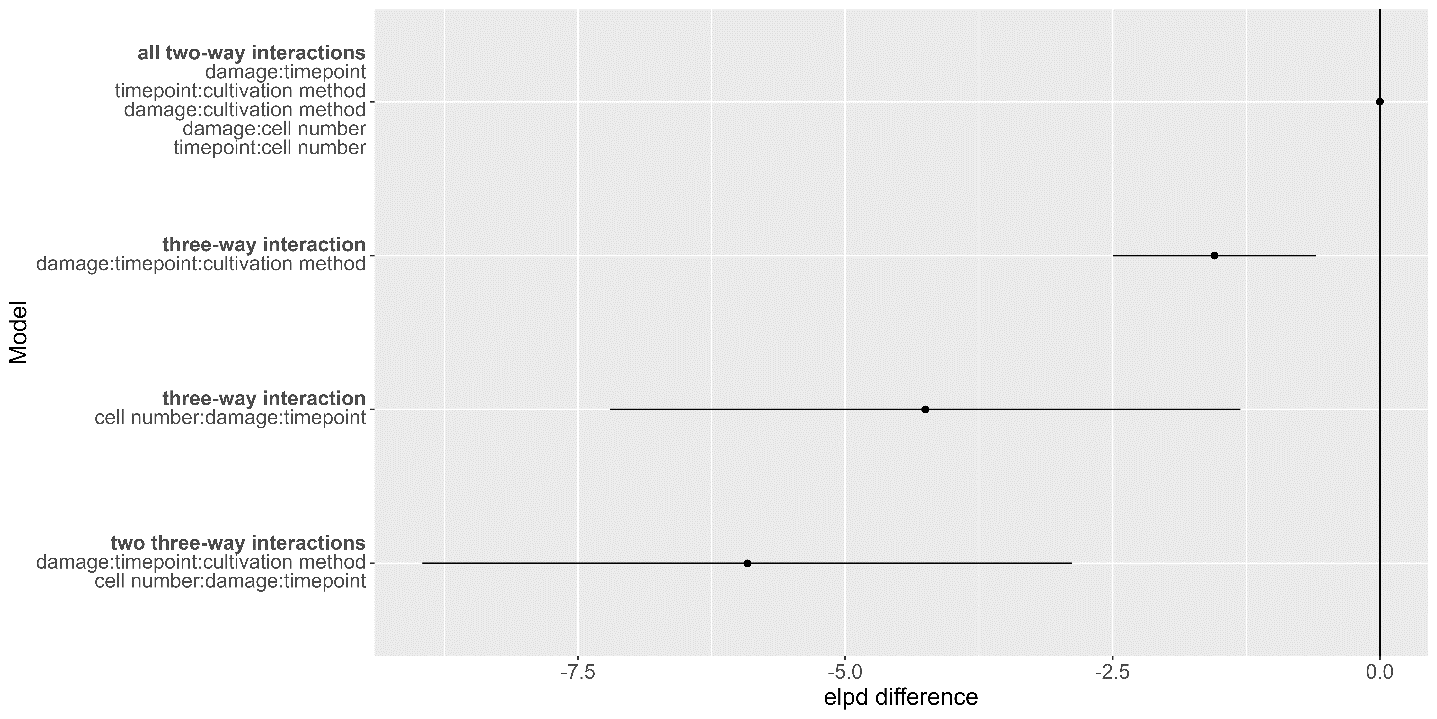


**Supplementary Figure** **3**: Graphical representations of elpd difference score and standard error for the comparison of the fitted models with three-way interactions or all resulting two-way interactions using brms package and loo_compare function.

**Supplementary Table** **4**: elpd difference score and standard error of the comparison of the fitted model with or without different two-way interactions using brms package and loo_compare function. all two-way refers to the interactions: damage:timepoint, timepoint:cultivation method, damage:cultivation method, damage:cell number, timepoint:cell number.

| Model | elpd_diff | se_diff |
| --- | --- | --- |
| all two-way interactions without cell number:damage | 0.0 | 0.0 |
| all two-way interactions | -2.7 | 3.8 |
| all two-way interactions without damage:timepoint | -3.7 | 4.4 |
| all two-way interactions without timepoint:cultivation method | -8.0 | 5.1 |
| two-way interaction: cell number:timepoint | -8.4 | 4.9 |
| all two-way interactions without damage:cultivation method | -8.4 | 4.9 |
| two three-way interactions:  damage:timepoint:cultivation method  cell number:damage:timepoint | -8.7 | 5.2 |
| all two-way interactions without cell number:timepoint | -36.6 | 11.6 |
| without two-way interactions | -42.3 | 11.4 |
| intercept-only | -208.9 | 26.1 |

: indicates interaction of variables


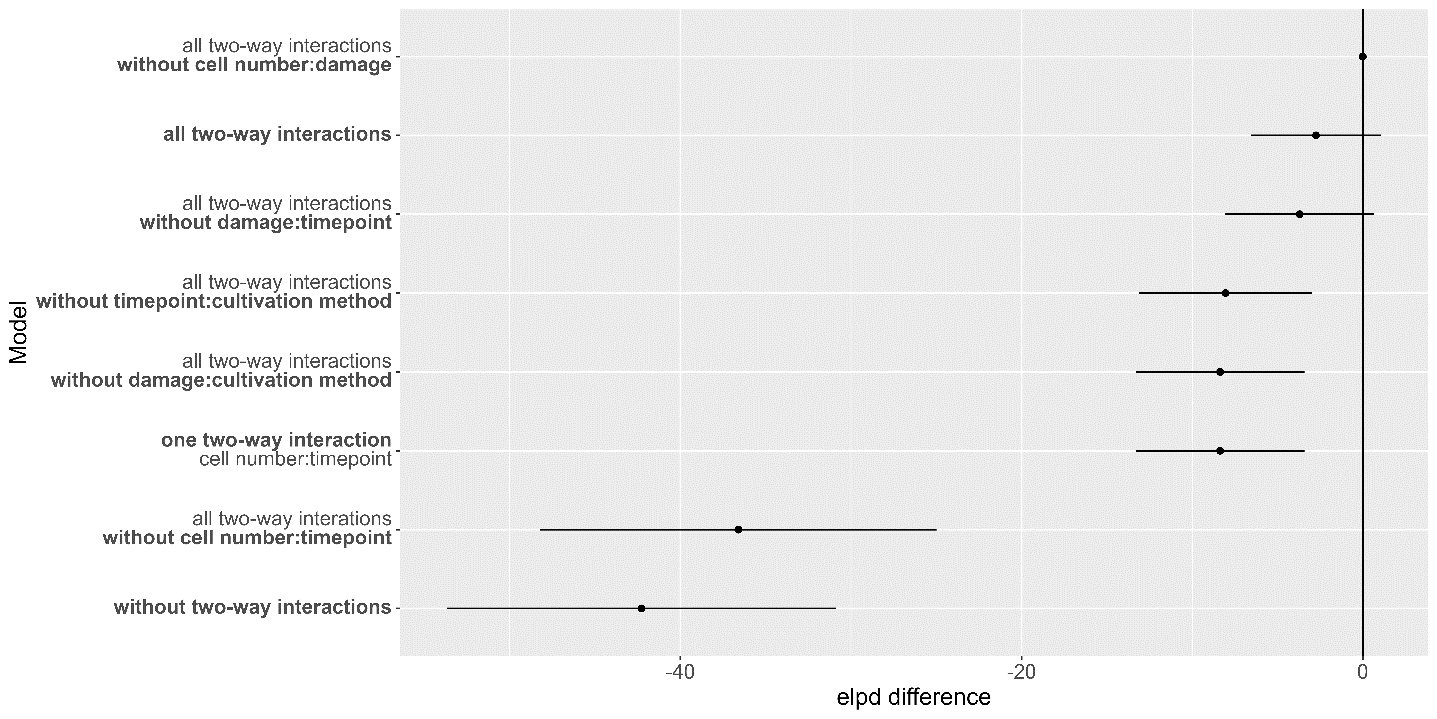


**Supplementary Figure** **4**: Graphical representation of elpd difference score and standard error of the comparison of the fitted model with different two-way interactions using brms package and loo_compare function. all two-way interactions refers to the interactions: damage:timepoint, timepoint:cultivation method, damage:cultivation method, damage:cell number, timepoint:cell number.

**Supplementary Table** **5**: Estimates, standard deviations (SD), and lower and upper limits of 95 % credible intervals (l-95 % CI, u-95 % CI) of the fitted model including the two-way interactions damage:timepoint, timepoint:cultivation method, damage:cultivation method, damage:cell number, timepoint:cell number.

| Parameter | Estimate | SD | l-95 % CI | u-95 % CI |
| --- | --- | --- | --- | --- |
| Intercept | 3.44 | 0.81 | 1.80 | 5.06 |
| cell number very low | 0.39 | 0.28 | -0.17 | 0.95 |
| cell number low | -0.17 | 0.15 | -0.46 | 0.12 |
| cell number high | 0.18 | 0.10 | -0.02 | 0.37 |
| cell number very high | 1.33 | 0.20 | 0.92 | 1.72 |
| Damaged | -0.28 | 0.08 | -0.43 | -0.12 |
| timepoint 2 | -0.22 | 0.21 | -0.65 | 0.19 |
| high-density cultivation method | -0.10 | 0.09 | -0.27 | 0.07 |
| cell number very low:damaged | 0.19 | 0.12 | -0.04 | 0.42 |
| cell number low:damaged | 0.17 | 0.10 | -0.02 | 0.36 |
| cell number high:damaged | 0.29 | 0.10 | 0.11 | 0.48 |
| cell number very high:damaged | 0.02 | 0.12 | -0.21 | 0.24 |
| cell number very low:timepoint 2 | -0.55 | 0.13 | -0.80 | -0.31 |
| cell number low:timepoint 2 | -0.15 | 0.10 | -0.34 | 0.03 |
| cell number high:timepoint 2 | 0.20 | 0.10 | 0.00 | 0.40 |
| cell number veryhigh:timepoint 2 | -0.61 | 0.11 | -0.83 | -0.38 |
| damaged:timepoint 2 | 0.11 | 0.07 | -0.04 | 0.25 |
| timepoint 2:high-density cultivation method | 0.02 | 0.10 | -0.19 | 0.21 |
| damaged:high-density cultivation method | 0.15 | 0.07 | 0.02 | 0.28 |
| sd(Intercept)* | 3.09 | 0.69 | 2.08 | 4.72 |
| sd(cell number verylow) | 0.95 | 0.23 | 0.60 | 1.49 |
| sd(cell number low) | 0.40 | 0.12 | 0.21 | 0.70 |
| sd(cell number high) | 0.18 | 0.09 | 0.02 | 0.39 |
| sd(cell number veryhigh) | 0.58 | 0.16 | 0.34 | 0.96 |
| sd(damaged) | 0.05 | 0.04 | 0.00 | 0.14 |
| sd(timepoint 2) | 0.68 | 0.18 | 0.41 | 1.11 |
| sd(high-density cultivation method) | 0.19 | 0.09 | 0.04 | 0.39 |
| sd(cell number very low:damaged) | 0.10 | 0.08 | 0.00 | 0.31 |
| sd(cell number low:damaged) | 0.08 | 0.06 | 0.00 | 0.23 |
| sd(cell number high:damaged) | 0.07 | 0.06 | 0.00 | 0.22 |
| sd(cell number very high:damaged) | 0.12 | 0.10 | 0.01 | 0.36 |
| sd(cell number very low:timepoint 2) | 0.16 | 0.12 | 0.01 | 0.45 |
| sd(cell number low:timepoint 2) | 0.07 | 0.06 | 0.00 | 0.23 |
| sd(cell number high:timepoint 2) | 0.12 | 0.09 | 0.00 | 0.34 |
| sd(cell number very high:timepoint 2) | 0.12 | 0.10 | 0.00 | 0.36 |
| sd(damaged:timepoint 2) | 0.09 | 0.07 | 0.00 | 0.26 |
| sd(timepoint 2:high-density cultivation method) | 0.25 | 0.12 | 0.03 | 0.53 |
| sd(damaged:high-density cultivation method) | 0.06 | 0.05 | 0.00 | 0.18 |

*sd standard deviations for random effects showing variation among genes


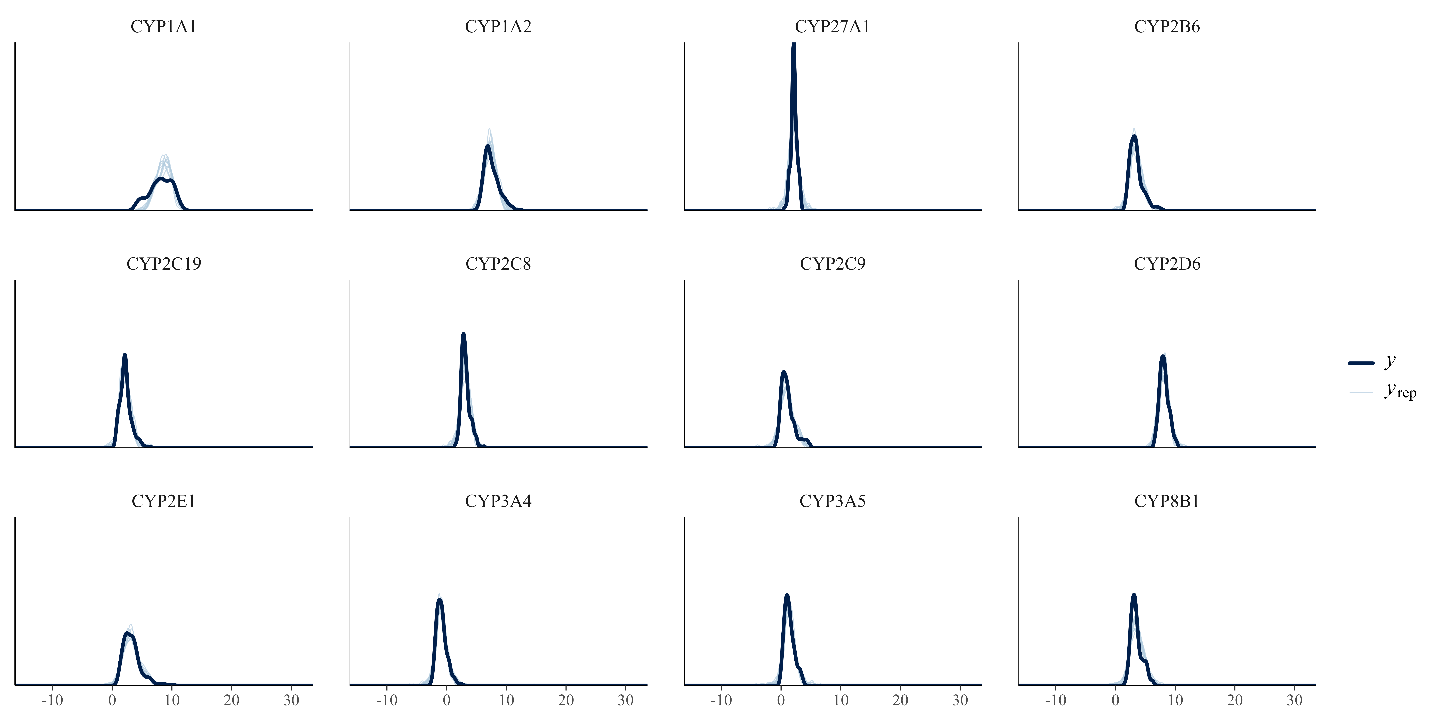


**Supplementary Figure 5**: Posterior predictive check of the type “dens_overlay_grouped” for the fitted model with two-way interactions: damage:timepoint, timepoint:cultivation method, damage:cultivation method, damage:cell number, timepoint:cell number.

**Supplementary Table** **6**: elpd difference score and standard error of the comparison of the fitted model from the data set excluding CYP1A1, CYP1A2 and CYP27A1 with or without different two-way interactions using brms package and loo_compare function. all two-way refers to the interactions: damage:timepoint, timepoint:cultivation method, damage:cultivation method, damage:cell number, timepoint:cell number.

| Model | elpd_diff | se_diff |
| --- | --- | --- |
| all two-way interactions without cell number:damage | 0.0 | 0.0 |
| all two-way interactions | -3.2 | 3.7 |
| two-way interaction cell number:timepoint | -3.7 | 4.2 |
| all two-way interactions without damage:cultivation method | -3.7 | 4.2 |
| all two-way interactions without damage:timepoint | -5.3 | 5.0 |
| all two-way interactions without timepoint:cultivation method | -6.1 | 4.9 |
| all two-way interactions without cell number:timepoint | -30.4 | 10.9 |
| without two-way interactions | -36.4 | 10.8 |
| intercept-only | -87.5 | 17.6 |

: indicates interaction of variables


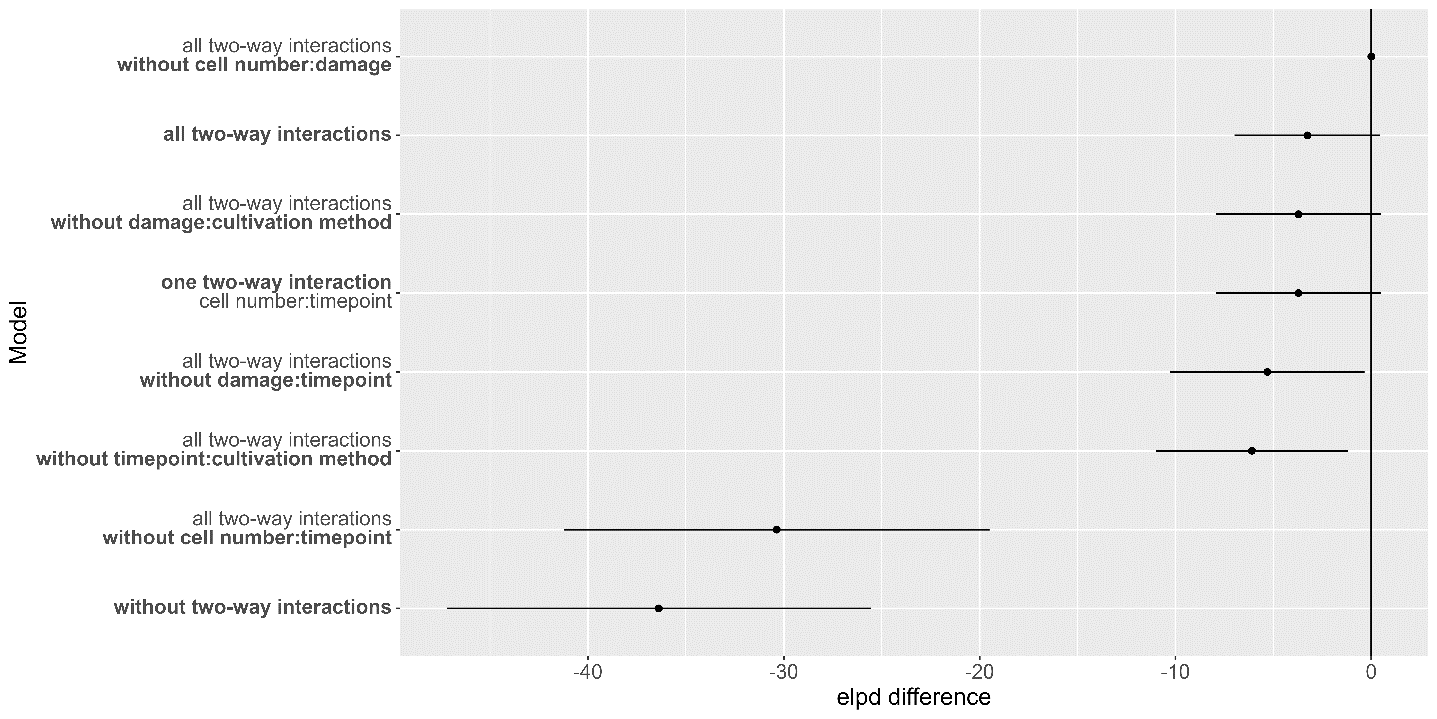


**Supplementary Figure** **6**: Graphical representation of elpd difference score and standard error of the comparison of the fitted model from the data set excluding CYP1A1, CYP1A2 and CYP27A1 with or without different two-way interactions using brms package and loo_compare function. all two-way interactions refers to the interactions damage:timepoint, timepoint:cultivation method, damage:cultivation method, damage:cell number, timepoint:cell number.

**Supplementary Table** **7**: Estimate, estimated error, and lower and upper limits of 95 % credible intervals (l-95 % CI, u-95 % CI) of the fitted model from the data set excluding CYP1A1, CYP1A2 and CYP27A1. The included two-way interactions are: damage:timepoint, timepoint:cultivation method, damage:cultivation method, damage:cell number, timepoint:cell number.

| Parameter | Estimate | Est.Error | l-95 % CI | u-95 % CI |
| --- | --- | --- | --- | --- |
| Intercept | 2.78 | 0.83 | 1.12 | 4.40 |
| cell number very low | 0.84 | 0.16 | 0.52 | 1.14 |
| cell number low | 0.00 | 0.10 | -0.21 | 0.20 |
| cell number high | 0.22 | 0.14 | -0.06 | 0.49 |
| cell number very high | 1.44 | 0.26 | 0.88 | 1.93 |
| damaged | -0.28 | 0.09 | -0.45 | -0.11 |
| timepoint 2 | -0.57 | 0.15 | -0.86 | -0.27 |
| high-density cultivation method | -0.14 | 0.10 | -0.33 | 0.06 |
| cell number very low:damaged | 0.14 | 0.14 | -0.12 | 0.41 |
| cell number low:damaged | 0.14 | 0.11 | -0.07 | 0.37 |
| cell number high:damaged | 0.26 | 0.11 | 0.05 | 0.46 |
| cell number very high:damaged | -0.07 | 0.13 | -0.31 | 0.19 |
| cell number very low:timepoint 2 | -0.60 | 0.15 | -0.88 | -0.31 |
| cell number low:timepoint 2 | -0.18 | 0.11 | -0.39 | 0.03 |
| cell number high:timepoint 2 | 0.16 | 0.11 | -0.05 | 0.38 |
| cell number very high:timepoint 2 | -0.61 | 0.12 | -0.85 | -0.36 |
| damaged:timepoint 2 | 0.21 | 0.08 | 0.06 | 0.36 |
| timepoint 2:high-density cultivation method | 0.16 | 0.10 | -0.04 | 0.36 |
| damaged:high-density cultivation method | 0.14 | 0.08 | -0.01 | 0.30 |
| sd(Intercept)* | 2.66 | 0.73 | 1.65 | 4.44 |
| sd(cell number very low) | 0.29 | 0.16 | 0.04 | 0.64 |
| sd(cell number low) | 0.13 | 0.10 | 0.01 | 0.37 |
| sd(cell number high) | 0.31 | 0.13 | 0.12 | 0.62 |
| sd(cell number very high) | 0.69 | 0.23 | 0.38 | 1.28 |
| sd(damaged) | 0.05 | 0.04 | 0.00 | 0.16 |
| sd(timepoint 2) | 0.35 | 0.12 | 0.17 | 0.65 |
| sd(high-density cultivation method) | 0.19 | 0.11 | 0.03 | 0.45 |
| sd(cell number very low:damaged) | 0.13 | 0.11 | 0.00 | 0.41 |
| sd(cell number low:damaged) | 0.10 | 0.08 | 0.00 | 0.30 |
| sd(cell number high:damaged) | 0.09 | 0.07 | 0.00 | 0.27 |
| sd(cell number very high:damaged) | 0.13 | 0.11 | 0.00 | 0.40 |
| sd(cell number very low:timepoint 2) | 0.18 | 0.14 | 0.01 | 0.51 |
| sd(cell number low:timepoint 2) | 0.08 | 0.07 | 0.00 | 0.26 |
| sd(cell number high:timepoint 2) | 0.11 | 0.09 | 0.00 | 0.32 |
| sd(cell number very high:timepoint 2) | 0.12 | 0.11 | 0.00 | 0.39 |
| sd(damaged:timepoint 2) | 0.07 | 0.06 | 0.00 | 0.22 |
| sd(timepoint 2:high-density cultivation method) | 0.16 | 0.13 | 0.01 | 0.46 |
| sd(damaged:high-density cultivation method) | 0.07 | 0.06 | 0.00 | 0.21 |

*sd standard deviations for random effects showing variation among genes
